# Supplementary material for: Tear Proteomics in Children and Adolescents with Type 1 Diabetes: A Promising Approach to Biomarker Identification of Diabetes Pathogenesis and Complications
Source: Int J Mol Sci. 2024 Sep 17;25(18):9994. doi: 10.3390/ijms25189994 (PMC11432293; doi:10.3390/ijms25189994)
Supplement: Supplementary file 1 [file ijms-25-09994-s001.zip › Table S1.pdf]

Table S1. Clinical and laboratory data of children with T1D depending on the presence of DKA at diagnosis			
	**DKA at diagnosis (n=25)**	**No DKA at diagnosis (n=29)**	**p-value**
Median age (years)	11.4 (9.4, 12.9)	11.6 (10.1, 13.0)	0.755
Sex (Male/female)	11/14	12/17	1.000
Pubertal/ prepubertal stage (n)	17/8	19/10	1.000
Median BMI SDS	0.67 (-0.02, 1.51)	0.68 (-0.32, 1.50)	0.822
Median diabetes duration (years)	2.1 (1.4, 5.1)	2.1 (1.5, 4.0)	0.945
Median age of onset (years)	8.1 (4.4, 10.9)	8.3 (6.7, 10.0)	0.400
Severity of DKA (mild/moderate-severe) (n)	13/12	-	-
Median HbA1C at diagnosis (mmol/mol, %)	113 (105, 133)<br>12.5% (11.8%, 14.3%)	101(83, 127)<br>11.4% (9.7%, 13.8%)	0.082
Median C- peptide at diagnosis (nmol/l)	0.14 (0.1, 0.2)	0.29 (0.15, 0.6)	**0.007**
Median HbA1c (mmol/mol, %)	57(54, 66)<br>7.4% (7.1%, 8.2%)	61(53, 68)<br>7.7% (7%, 8.3%)	0.794
Median glucose(mg/dl)	170 (138, 200)	159 (127, 199)	0.630
Median total cholesterol (mg/dl)	171 (142, 187)	160 (144, 179)	0.682
Median LDL (mg/dl)	94 (75, 111)	79 (67, 98)	0.236
Median triglycerides (mg/dl)	54 (42, 67)	52 (40, 66)	0.504
Pump therapy/MDI	4/21	3/26	0.833
Usage of FGM-CGM (yes/no)	22/3	23/6	0.625
Median TIR (%)	58 (53, 68)	61 (45, 74)	0.990
Median TBR (%)	2 (1, 4)	2 (1, 4)	0.414

Median TAR (%)	38 (28, 43)	37 (26, 52)	0.715
Median CV (%)	37 (33, 38)	36 (32, 42)	0.822
Episodes of SH (n)	none	3	0.290

Values are expressed as mean ((±SD) or median (25th and 75th percentiles) for continuous variables and as absolute numbers(n) and frequencies for categorical variables. BMI SDS, Body Mass Index standard deviation score; CGM, continuous glucose monitoring, CV, coefficient of variation; DKA, diabetic ketoacidosis, FGM, flash glucose monitoring; MDI, multiple daily injections, SH: severe hypoglycemia; TBR, Time Below Target Range; TIR, Time In Target Range; TAR, Time Above Target Range; T1D, type 1 diabetes
