# Supplementary material for: Tear Proteomics in Children and Adolescents with Type 1 Diabetes: A Promising Approach to Biomarker Identification of Diabetes Pathogenesis and Complications
Source: Int J Mol Sci. 2024 Sep 17;25(18):9994. doi: 10.3390/ijms25189994 (PMC11432293; doi:10.3390/ijms25189994)
Supplement: Supplementary file 1 [file ijms-25-09994-s001.zip › Table S2.pdf]

Table S2. Clinical and laboratory data of children with T1D depending on their glycemic control			
	**Good glycemic control [HbA1c≤56 mmol/mol (7.3%)] (n=24)**	**Poor glycemic control [HbA1c >56 mmol/mol (7.3%)] (n=32)**	**p-value**
Median age(years)	11.0 (8.5, 12.3)	12.0 (10.8, 14.0)	0.054
Sex (Male/female)	12/12	13/19	0.590
Pubertal/ prepubertal stage	12/12	25/7	**0.045**
Median BMI SDS	0.51 (-0.29, 1.56)	0.68 (-0.11, 1.40)	0.645
Median diabetes duration (years)	2 (1.6, 3.8)	2.4 (1.6, 5.3)	0.362
Median age at T1D onset (years)	7.1 (6.3, 9.7)	9.1 (5.7, 11.0)	0.371
Median HbA1c (mmol/mol, %)	52 (49, 54),<br>6.9% (6.6%, 7.1%)	64 (61, 70)<br>8% (7.7%, 8.6%)	**<0.001**
Median glucose (mg/dl)	137 (118, 178)	185 (153, 246)	**0.002**
Median total cholesterol (mg/dl)	160 (139, 179)	171 (150, 188)	0.180
Median LDL (mg/dl)	78 (67, 103)	87 (74, 113)	0.278
Median triglycerides (mg/dl)	52 (41, 60)	54 (42, 71)	0.339
Pump therapy /MDI	2/22	5/27	0.683
Usage of FGM-CGM (yes/no)	20/4	25/7	0.884
Median TIR (%)	68 (58, 80)	53 (43, 61)	**<0.001**
Median TBR (%)	2 (1, 4)	2 (1, 4)	0.456
Median TAR (%)	28 (18, 38)	42 (32, 55)	**0.001**
Median CV (%)	35 (32, 38)	38 (32, 42)	0.187
Episodes of SH (n)	none	3	0.346

Values are expressed as mean ((±SD) or median (25th and 75th percentiles) for continuous variables and as absolute numbers(n) and frequencies for categorical variables. BMI SDS, Body Mass Index standard deviation score; CGM, continuous glucose monitoring, CV, coefficient of variation; FGM, flash glucose monitoring; MDI, multiple daily injections, SH: severe hypoglycemia; TBR, Time Below Target Range; TIR, Time In Target Range; TAR, Time Above Target Range; T1D, type 1 diabetes
